# Increases in the rates of primary and revision knee replacement are reducing: a 15-year registry study across 3 continents

**DOI:** 10.1080/17453674.2020.1749380

**Published:** 2020-04-14

**Authors:** Peter L Lewis, Stephen E Graves, Otto Robertsson, Martin Sundberg, Elizabeth W Paxton, Heather A Prentice, Annette W-Dahl

**Affiliations:** a Australian Orthopaedic Association National Joint Replacement Registry, Adelaide, Australia;;; b Swedish Knee Arthroplasty Register, Lund, Sweden;; c Surgical Outcomes and Analysis, Kaiser Permanente, San Diego, CA, USA;; d Lund University, Faculty of Medicine, Clinical Sciences Lund, Department of Orthopedics, Lund, Sweden

## Abstract

Background and purpose — Rates of knee replacement (KR) are increasing worldwide. Based on population and practice changes, there are forecasts of a further exponential increase in primary knee replacement through to 2030, and a corresponding increase in revision knee replacement. We used registry data to document changes in KR over the past 15 years, comparing practice changes across Sweden, Australia, and the United States. This may improve accuracy of future predictions.

Patients and methods — Aggregated data from the Swedish Knee Arthroplasty Register (SKAR), the Australian Orthopaedic Association National Joint Replacement Registry (AOANJRR), and the Kaiser Permanente Joint Replacement Registry (KPJRR) were used to compare surgical volume of primary and revision KR from 2003 to 2017. Incidence was calculated using population census statistics from Statistics Sweden and the Australian Bureau of Statistics, as well as the yearly active membership numbers from Kaiser Permanente. Further analysis of KR by age < 65 and ≥ 65 years was carried out.

Results — All registries recorded an increase in primary and revision KR, with a greater increase seen in the KPJRR. The rate of increase slowed during the study period. In Sweden and Australia, there was a smaller increase in revision surgery compared with primary procedures. There was consistency in the mean age at surgery, with a steady small decrease in the proportion of women having primary KR. The incidence of KR in the younger age group remained low in all 3 registries, but the proportional increases were greater than those seen in the ≥ 65 years of age group.

Interpretation — There has been a generalized deceleration in the rate of increase of primary and revision KR. While there are regional differences in KR incidence, and rates of change, the rate of increase does not seem to be as great as previously predicted.

Knee replacement (KR) has a favorable survival rate with cumulative revision as low as 3% at 10 years (AOANJRR 2018, SKAR 2018) and this result appears to be improving with time as wear-related revisions become less common (Sharkey et al. 2014, Koh et al. 2017, Postler et al. 2018).

Throughout the last decade, national joint replacement registries have recorded increasing yearly volumes of KR (AOANJRR 2018, NJR 2018, SKAR 2018). The reasons for this increase in procedure numbers are proposed to be increased surgeon and patient acceptance of KR (Hamilton et al. 2015), improved longevity (Patel et al. 2015), increasing incidence of osteoarthritis (OA), and use of KR in younger patients (Weinstein et al. 2013, Leyland et al. 2016, Karas et al. 2019).

With increasing primary KR use it is predicted that the numbers of revision procedures will also rise (Kumar et al. 2015, Patel et al. 2015). Not only are more people receiving a KR, but some of the factors driving increased primary usage of KR also contribute to increased failure. These include longer life-expectancy, whereby patients with a KR have more time to be revised, and use in young and obese patients who place higher demands on their KR (Hamilton et al. 2015). Counter-balancing this trend, to a small extent, is improved prosthesis performance (Pitta et al. 2018).

There is international variation in the use of KR (Kurtz et al. 2011). In a comparative study of 18 countries in 2008, Kurtz et al. (2011) found a range of 8.6 to 213 primary procedures/100,000 population, and a range of 0.2 to 28 revision procedures/100,000 population, but they could not determine if the observed variation related to healthcare systems, access to care, number and distribution of orthopedic surgeons, or the prevalence of joint disease. There are expectations of exponential increases for both primary and revision KR. However, predictions of revision KR in the year 2030 compared with 2005 levels vary widely, from a 75% increase in Taiwan to a 600% increase in the USA and a similar increase in the UK (Kurtz et al. 2007, Kumar et al. 2015, NJR 2018). A further study comparing 24 OECD countries’ KR utilization predicted a 400% increase by 2030 (Pabinger et al. 2015). There are other predictive models with a more conservative forecast for the United States (Inacio et al. 2017).

We performed a multi-country comparison of KR, comparing the changing procedure volume and incidence of primary and revision KR using data from the Swedish Knee Arthroplasty Register (SKAR), the Australian Orthopaedic Association National Joint Replacement Registry (AOANJRR), and the Kaiser Permanente Joint Replacement Registry (KPJRR) over a 15 year period (2003–2017).

## Patients and methods

Data were obtained for the period January 1, 2003 until December 31, 2017 for KR procedures recorded in the SKAR, AOANJRR, and the KPJRR. Primary KR procedures were defined as all initial unicompartmental, patellofemoral, and total KR. Where replacements were bilateral, both knees were included. Revision KR included all revision procedures of a previous replacement (partial or total) where 1 or more components were added, removed, or exchanged, regardless of whether this was the 2nd or subsequent procedure in chronology. The capture rate of these registries exceeds 95% and loss to follow-up was less than 8% over the study period. Validation and quality control methods of these registries have been published previously (Paxton et al. 2010, Robertsson et al. 2014, AOANJRR 2018).

There were 1,133,079 KR included in this analysis. The SKAR contributed 199,020 KR (186,473 primary and 12,547 revision procedures), there were 732,521 KR from the AOANJRR (674,045 primary and 58,476 revision procedures), and 201,350 KR from the KPJRR (188,538 primary and 12,812 revision procedures) (Table 1).

**Table 1.** Yearly totals of knee replacement (KR) procedures recorded in the SKAR, AOANJRR, and KPJRR

KR type **^a^**	2003	2004	2005	2006	2007	2008	2009	2010	2011	2012	2013	2014	2015	2016	2017
**Sweden**															
Primary	8,832	9,195	9,797	10,691	10,527	11,004	12,841	12,848	12,845	13,411	13,361	13,145	12,924	14,053	14,964
Uni	982	892	928	916	728	712	693	689	594	536	494	465	648	984	1169
PF	11	16	21	9	12	17	37	31	52	43	56	58	65	52	48
Total	7,339	8,287	8,848	9,766	9,787	10,275	12,111	12,228	12,198	12,832	12,808	12,622	12,206	13,008	13,743
Revision	596	625	650	650	657	702	758	860	845	869	1002	959	936	934	945
All	9,428	9,820	10,447	11,341	11,184	11,706	13,599	13,708	13,690	14,280	14,363	14,104	13,860	14,987	15,909
**Australia**															
Primary	26,008	7,540	30,409	31,231	33,064	36,160	37,683	40,838	43,051	44,839	46,903	49,813	53,578	55,878	59,002
Uni	4,109	3,730	3,382	3,628	2,502	3,225	3,087	2,615	2,411	21,46	2,137	2,270	2,557	3,056	3,652
PF	151	180	174	181	195	232	229	268	247	225	246	234	248	307	298
Total	21,735	23,603	26,337	27,376	29,294	32,622	34,307	37,922	40,375	42,453	44,495	47,288	50,763	52,510	55,077
Revision	2,314	2,663	2,721	2,826	2,994	3,250	3,294	3,716	3,894	3,910	4,173	4,301	4,447	4,559	4,791
All	28,322	30,203	33,130	34,057	36,058	39,410	40,977	44,554	46,945	48,749	51,076	54,114	58,025	60,437	63,793
**Kaiser Permanente**															
Primary	4,271	5,824	7,050	8,255	9,283	10,234	10,806	12,904	13,495	14,084	15,445	17,796	18,324	20,093	20,672
Uni	144	234	210	212	200	330	448	420	371	439	522	631	602	563	579
PF	7	6	6	14	10	24	27	35	38	30	44	57	54	65	84
Total	4,120	5,584	6,834	8,029	9,073	9,880	10,331	12,449	13,086	13,616	14,879	17,109	17,669	19,465	20,009
Revision	274	363	456	556	627	773	766	850	981	1,021	1,091	1,173	1,267	1,305	1,309
All	4,545	6,187	7,506	8,810	9,910	11,007	11,572	13,754	14,476	15,106	16,536	18,969	19,592	21,398	21,981

**^a^**Uni = unicompartmental; PF = patellofemoral

Note: A small number of other primary knee replacement (unispacer, partial resurfacing, bicompartmental) are included in primary knee totals.

### Statistics

Aggregated data regarding type of procedure as well as patient age and sex were obtained. Population data were obtained from Statistics Sweden and the Australian Bureau of Statistics, as well as the yearly active membership numbers from Kaiser Permanente.

Comparisons were made between countries for yearly procedure volume for both primary and revision KR, as well as yearly incidence per 100,000 population. Stratified analysis for ages < 65 and ≥ 65 years was also carried out. Inclusion of bilateral procedures and multiple revisions was thought to affect each country’s analysis similarly. Mean age and sex tables were compiled and the proportions by ages < 65 and ≥ 65 years for both primary and revision KR were included.

Annual percentage change in procedure volume for both primary and revision KR was calculated and the mean for each of the 3 5-year time periods was derived, as described by Patel et al. (2015), to summarize trends in these procedures over time and across countries.

### Ethics, funding, and conflicts of interest

Ethics approval covering the SKAR data use was approved by the Ethics Board of Lund University (LU20-02). The AOANJRR is a declared Commonwealth of Australia Quality Assurance Activity under section 124X of the Health Insurance Act, 1973. All AOANJRR studies are conducted in accordance with ethical principles of research (Helsinki Declaration II). Approval for inclusion of data from the Kaiser Permanente Joint Replacement Registry Institutional Review Board approval (#5488) was granted on November 15, 2018.

There was no funding. There are no conflicts of interest.

## Results

**Figure 1.**
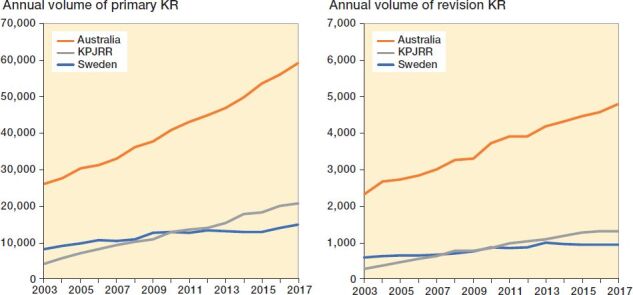
Yearly procedure volume of primary KR (left panel) and revision KR (right panel) recorded by the SKAR, AOANJRR, and KPJRR from 2003 to 2017.

Throughout the 15 years from 2003 to 2017, annual primary KR procedure volume increased from 8,832 in 2003 to 14,964 in 2017 in Sweden, from 26,008 to 59,002 in Australia, and from 4,271 to 20,672 in the KPJRR. The proportion of total KR rose in both Sweden and Australia from 83.1% and 83.6% to 91.8% and 93.3%, respectively, while the volume of unicompartmental KR reduced. This contrasts with the KPJRR, which had a more constant proportion of total KR remaining around 96% for the entire period. In all 3 registries, the proportion of patellofemoral KR remained low (less than 1%). Over the study period, revision KR procedure volume increased from 596 in Sweden to 945, from 2,314 in Australia to 4,791, and from 274 in the KPJRR to 1,309 (Figure 1). Primary KR volume increases were 79% in Sweden, 127% in Australia, and 384% in the KPJRR. During the same time period, revision KR procedure volume increases were 59% in Sweden, 107% in Australia, and 378% in the KPJRR.

**Figure 2.**
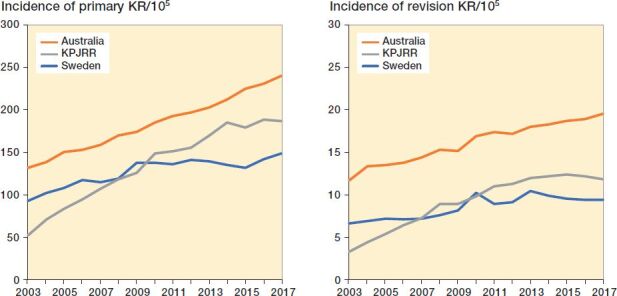
Yearly incidence of primary KR (left panel) and revision KR (right panel) per 10^5^ population recorded by the SKAR, AOANJRR, and KPJRR from 2003 to 2017.

**Figure 3.**
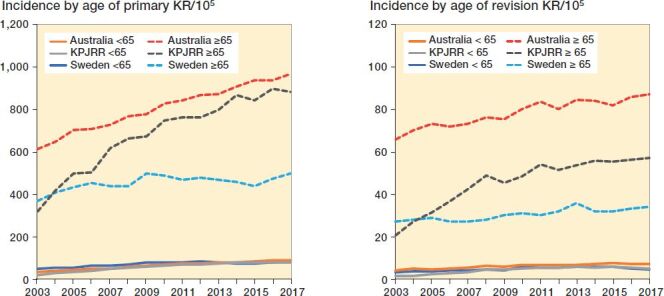
Yearly incidence of primary KR (left panel) and revision KR (right panel) by patient age < 65 and ≥ 65 years per 10^5^ population recorded by the SKAR, AOANJRR, and KPJRR from 2003 to 2017.

The incidence of primary KR per 100,000 population over this same time span in Sweden increased from 73 to 131 and revision KR incidence increased from 6.6 to 9.4, while in Australia primary KR incidence rose from 132 to 240 and revision KR incidence increased from 11.7 to 19.5. In the KPJRR cohort primary KR incidence/10^5^ insured increased from 52 to 187 and revision KR from 3.3 to 11.8 (Figure 2). By this measure, primary KR incidence increased from 2003 to 2017 by 79% in Sweden, 102% in Australia, and 258% in the KPJRR, while over this same time revision KR incidence increased by 42% in Sweden, 63% in Australia, and 255% in the KPJRR. When stratified by age < 65 and ≥ 65 years, the annual incidence/10^5^ population for the younger group remained less than 90 for primary KR and less than 8 for revision KR in all 3 registries, while the older cohort from the KPJRR showed the largest increases (from 320 to 884 for primary KR and from 21 to 57 for revision KR) (Figure 3). While the incidence/10^5^ in the younger age group remained low, the proportional change over the 15 years in this group for primary KR was 76%, 141%, and 276% for Sweden, Australia, and the KPJRR, respectively, while it was 35%, 58%, and 177% for the ≥ 65 years age group. Over the same time period the increases for revision KR incidence for the < 65 years age group were 39%, 85%, and 277%, and for the ≥ 65 years age group 26%, 32%, and 171% in Sweden, Australia, and the KPJRR, respectively.

**Figure 4.**
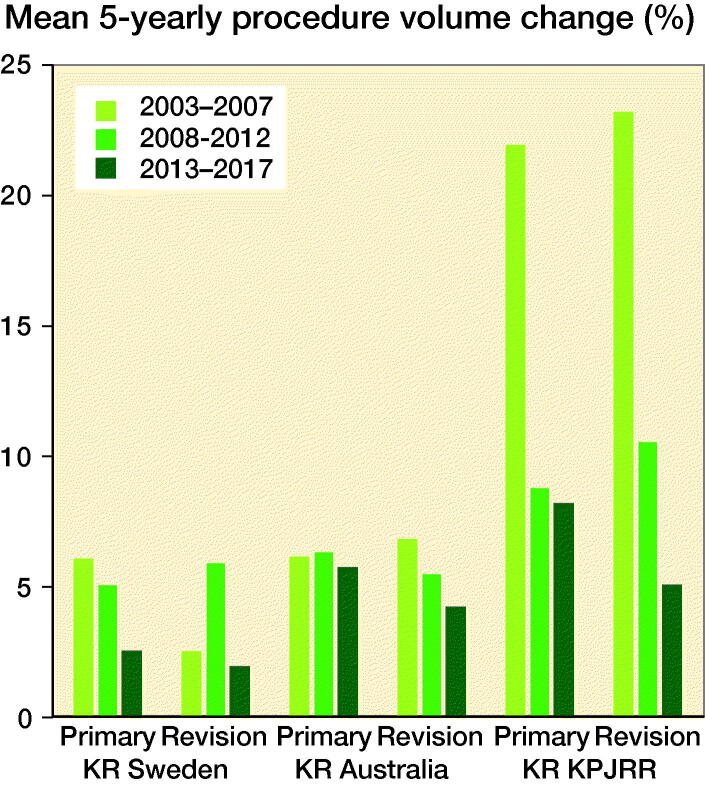
Mean 5-yearly percentage increases in procedure volume in SKAR, AOANJRR, and KPJRR.

When the mean change for each of the 3 5-year periods was calculated for primary and revision KR, all regions showed a deceleration in the increase. The exception is an increase in revision in Sweden between the periods 2003–2007 to 2008–2012 (Figure 4).

During the study period, the mean age of primary and revision KR patients remained stable in all countries (Table 2). The proportion of patients aged < 65 years for both primary and revision KR varied in a narrow range for each registry, peaking in the years 2008–2012 and decreasing again in all instances toward the end of the study period (Table 2). The proportion of women undergoing primary KR decreased in all countries over this 15-year period. The proportion of women undergoing revision KR was lower than in primary KR in all countries and showed little change with time (Table 2).

**Table 2.** Yearly mean ages, percentage women, and proportion age < 65 years for primary and revision KR by registry

Factor	2003	2004	2005	2006	2007	2008	2009	2010	2011	2012	2013	2014	2015	2016	2017
**Sweden**															
Primary KR															
mean age	69.6	69.5	69.6	69.3	69.3	68.9	69.0	68.8	68.7	68.6	68.6	68.8	68.6	68.9	68.9
women (%)	61.0	61.9	60.0	59.5	60.0	59.3	58.3	58.4	58.2	57.7	57.0	57.2	57.0	56.0	55.7
age < 65 (%)	31.3	31.1	31.0	33.1	32.9	34.3	34.4	34.1	34.7	34.7	33.9	32.9	33.9	33.7	33.2
Revision KR															
mean age	70.7	70.5	70.5	69.9	69.9	69.8	69.2	68.7	68.5	69.4	68.8	68.3	68.5	69.4	69.9
women (%)	58.2	61.0	59.2	61.5	57.5	54.0	61.1	56.6	56.1	55.5	55.5	51.9	54.2	54.6	53.2
age < 65 (%)	29.0	28.9	30.9	35.1	34.2	33.7	33.6	36.7	36.1	33.3	33.5	35.9	34.6	30.9	27.9
**Australia**															
Primary KR															
mean age	68.7	68.7	68.8	68.6	68.4	68.2	68.1	68.0	67.9	68.1	68.0	68.0	67.9	67.8	67.9
women (%)	56.3	56.8	57.2	56.6	56.9	56.5	56.5	56.3	55.9	56.1	56.4	55.7	55.4	55.5	54.8
age < 65 (%)	32.2	32.4	32.7	33.5	34.2	35.6	35.9	36.2	36.7	35.2	35.6	34.8	35.1	35.4	34.5
Revision KR															
mean age	69.9	68.8	69.5	69.0	69.1	68.7	68.9	68.4	68.6	68.5	68.7	68.6	68.2	68.9	68.9
women (%)	51.6	51.8	50.5	51.5	52.7	51.9	50.8	52.2	50.7	52.3	51.2	52.4	51.3	50.0	51.1
age < 65 (%)	28.7	32.5	29.7	32.4	33.0	34.4	33.9	35.5	33.8	34.2	32.8	34.6	31.3	31.3	30.3
**Kaiser Permanente**															
Primary KR															
mean age	67.8	67.8	67.8	67.7	67.1	67.1	67.0	67.3	67.3	67.3	67.3	67.3	67.3	67.4	67.5
women (%)	64.4	63.0	64.6	63.4	62.4	61.2	61.7	60.9	61.1	60.6	61.0	60.2	61.5	60.9	60.3
age < 65 (%)	35.8	37.3	36.8	37.2	40.5	41.5	41.3	39.9	40.7	38.8	38.4	38.4	38.0	36.9	36.9
Revision KR															
mean age	68.0	68.0	67.6	66.2	67.5	66.7	66.4	67.3	66.7	66.7	67.1	67.2	66.9	67.5	67.7
women (%)	48.9	54.5	53.3	55.5	53.9	52.8	58.9	55.5	53.0	57.9	53.4	56.9	56.9	53.8	54.8
age < 65 (%)	35.4	34.7	37.9	41.1	39.9	43.9	44.1	43.4	42.1	43.2	41.4	39.7	41.8	39.4	35.7

## Discussion

Through the last 15 years, primary and revision KR have increased in all 3 countries studied. Suggested reasons for this widespread change are the increase in the prevalence of knee OA, or increased recognition of the utility of KR by surgeons and the community (Weinstein et al. 2013, Hamilton et al. 2015). The growth in KR in the KPJRR was greater than that seen in the other 2 registries with no clear reason for this difference. This may indicate a previously unmet demand is being filled in this population or be due to local market conditions in the USA. A previous population predictive study has suggested that the rising rate of KR is linked to increasing community obesity (Culliford et al. 2015).

As population profiles may vary both between countries and over time, perhaps a better measure for comparison is incidence/10^5^ population. Australia has a higher incidence of both primary and revision KR/10^5^ but the incidence in the KPJRR is approaching that of Australia in primary KR. Incidence changes show a parallel increase in primary and revision KR in the KPJRR, while revision incidence growth in both Sweden and Australia has been proportionately less than that of primaries. Incidence increases in the KPJRR cohort were less than the changes in procedure numbers, indicating a larger rise in the population with this insurance.

There has been little change in the mean age of patients receiving either a primary or revision KR in all countries, and little variation in the proportions of KR for patients aged < 65 years. Increases in the use of KR in younger patients are therefore balanced by a comparable increase in the ≥ 65 years age group. This counters the suggestion that KR has been proportionally more frequently used for younger patients over this time (Karas et al. 2019). While the proportion of younger to older KR patients remained stable, the percentage increases in incidence/10^5^ in the younger group were greater, a finding consistent with others (Weinstein et al. 2013, Pabinger et al. 2015). In all 3 registries over the study period, there is an increase in the proportion of males receiving a primary KR, and as there are proportionately more males requiring revision (Table 2) this trend may increase future revision rates.

Variation in rates of KR among countries may be due to local economic concerns and health policy, differences in rates of OA, availability of pre-surgical treatments for OA, and access to KR, as well as surgeon availability and variation in thresholds for suitability for operative treatment. The higher incidence of revision KR in Australia compared with the other countries may simply mirror the higher incidence of primary surgery or be due to differences in surgical practice (such as the proportional use of patella resurfacing or cementless fixation) but could also be related to less restricted prosthesis choice in this country.

Part of the reason for a smaller rate of increase in revision KR when compared with primary KR in Sweden and Australia may be due to the decrease in proportion of unicompartmental KR in these countries, as partial KR has more than 2.5 times the rate of revision of total KR at 10 years (AOANJRR 2018). This change may also reflect improved prosthesis performance during this time span, related to factors such as the introduction of more component sizing options or highly cross-linked polyethylene (de Steiger et al. 2015, Turnbull et al. 2016). Alternatively, the relative slowing of revision compared with primary KR may be due to the presence of a time lag between increased numbers of primaries and when they will require revision.

When analysis was carried out by 5-year time periods the increase in both primary and revision surgery decelerated in all countries over the duration of this study, with the only exception being the increase in revision KR in Sweden in 2008–2012 when compared with the earlier period. From our findings, we contend that previous studies predicting an exponential increase in primary and revision KR are incorrect and that a universal deceleration of the growth in primary KR has been experienced, with an even greater slowing in growth of revision KR being evident (Kurtz et al. 2007, Kumar et al. 2015, Patel et al. 2015). However, there is quite a large variation between countries, with the KPJRR cohort showing the greatest percentage increase in both of these procedure types, while Australia and Sweden have a lower growth rate and increasing disparity between primary and revision rates with time. The variations between countries seen in this study over this time period show that predictive models of future demands for 1 region may not translate to others. Our findings also imply that more conservative future estimates would potentially be more accurate, as suggested by Inacio et al. (2017). While there has been a slowing of the increase in the rate of KR in all 3 countries, our findings may not be generalizable to other countries, where different health systems are in place. A limitation of our study is its retrospective nature, which may have little bearing on future trends. In addition, the study is a simple overview of population changes with time, which can be influenced by many factors, and little or no detail as to the reasons for changes is revealed. This area could be the subject of further analysis. Caution should be used in extrapolating the findings of the cohort from the KPJRR as these may not be representative of the changes found elsewhere in the United States. In addition, revision incidence would be overestimated as it has been calculated irrespective of multiple surgeries for the same patient or knee. These methodological limitations are expected to affect each registry similarly and be consistent throughout the study period. There may also be other unknown influences, such as the introduction of new technologies or changing health policies and economics, which can affect each country differently, and these have not been examined in this study.

## Conclusion

While there has been an increase in both primary and revision KR across all 3 countries during the past 15 years, the rate of increase has slowed. While there are regional differences in KR incidence, and also differences in rates of change, the rate of increase, particularly in Sweden and Australia, does not seem to be as great as previously predicted. Additionally, the rate of increase in revision KR in these 2 countries is less than the increase in primary KR.
